# Antibacterial effects of 18 medicinal plants used by the Khyang tribe in Bangladesh

**DOI:** 10.1080/13880209.2018.1446030

**Published:** 2018-03-13

**Authors:** Md Shahadat Hossan, Hassan Jindal, Sarah Maisha, Chandramathi Samudi Raju, Shamala Devi Sekaran, Veeranoot Nissapatorn, Fatima Kaharudin, Lim Su Yi, Teng Jin Khoo, Mohammed Rahmatullah, Christophe Wiart

**Affiliations:** a School of Pharmacy, University of Nottingham, Semenyih, Malaysia;; b Department of Medical Microbiology, University of Malaya, Kuala Lumpur, Malaysia;; c School of Allied Health Science, Walailak University, Thai Buri, Thailand;; d Department of Pharmacy, University of Development Alternative, Dhaka, Bangladesh

**Keywords:** Methicillin-resistant *Staphylococcus aureus*, cinnamaldehyde, vancomycin, bacterial resistance, eugenol, gallic acid, *Mentha arvensis*, *Terminalia bellirica*, *Cinnamomum cassia*

## Abstract

**Context:** The resistance of bacteria to antibiotics is raising serious concern globally. Asian medicinal plants could improve the current treatment strategies for bacterial infections. The antibacterial properties of medicinal plants used by the Khyang tribe in Bangladesh have not been investigated.

**Objective:** The present study examines the antibacterial properties of 18 medicinal plants used by the Khyang tribe in day-to-day practice against human pathogenic bacteria.

**Materials and methods:** Leaves, bark, fruits, seeds, roots and rhizomes from collected plants were successively extracted with hexane, ethyl acetate and ethanol. The corresponding 54 extracts were tested against six human pathogenic bacteria by broth microdilution assay. The antibacterial mode of actions of phytoconstituents and their synergistic effect with vancomycin and cefotaxime towards MRSA was determined by time-killing assay and synergistic interaction assay, respectively.

**Results and discussion:** Hexane extract of bark of *Cinnamomum cassia* (L.) J. Presl. (Lauraceae) inhibited the growth of MRSA, *Enterococcus faecalis*, *Escherichia coli*, *Pseudomonas aeruginosa*, *Klebsiella pneumoniae* and *Acinetobacter baumannii* with MIC values below 100 µg/mL. From this plant, cinnamaldehyde evoked at 4 × MIC in 1 h an irreversible decrease of MRSA count Log10 (CFU/mL) from 6 to 0, and was synergistic with vancomycin for MRSA with fractional inhibitory concentration index of 0.3.

**Conclusions:** Our study provides evidence that the medicinal plants in Bangladesh have high potential to improve the current treatment strategies for bacterial infection.

## Introduction

The resistance of bacteria to antibiotics has increased to such extend that the World Health Organization (WHO) warns of a ‘post-antibiotic era’ (O’Neill 2014; WHO 2014). In 1998, 5% of *Escherichia coli* isolated from hospitals in the Netherlands were resistant to fluoroquinolones (Goettsch et al. 2000). In 2014, five out of six WHO regions were affected with 50% or more resistance of *Escherichia coli* to fluoroquinolone (WHO 2014). Carbapenem-resistant *Klebsiella pneumoniae* has first been reported in Scotland in the late nineties (MacKenzie et al. 1997). In 2005, 3.3% *Klebsiella pneumoniae* isolates were resistant to carbapenem in Brooklyn hospitals (Bratu et al. 2005). In 2014, two out of six WHO regions reported 50% or more resistance of *Klebsiella pneumoniae* to carbapenem (WHO 2014). Today *Acinetobacter baumannii* (Moraxellaceae) resists almost all known antibiotics (Peleg et al. 2008). The resistance of *Staphylococcus aureus* (Staphylococcaceae) to methicillin emerged in 1961 (Jevons 1961). Methicillin-resistant *Staphylococcus aureus* (MRSA) is now resistant to vancomycin and cefotaxime and poses a threat to human health (Fung-Tomc et al. 1988; Neu 1992; Stryjewski and Corey 2014).

In an attempt to control bacterial resistance, WHO recommends ‘to develop the economic case for sustainable investment that takes account of the needs of all countries and to increase investment in new medicines’ (WHO 2014). However, approval for new antibacterial agent by the FDA has been decreasing (Charles and Grayson 2004; Spellberg et al. 2004). According to Alanis (2005), the traditional antibiotic structures have been almost exhausted to the point that antibacterial research is literally crying for new chemical entities that could be found by using fresh and different research approaches. Medicinal plants in Asia have the ability to synthesize a fascinating array of low molecular weight molecules with structures completely unrelated to antibiotics. One example is the alkaloid berberine produced by *Tinospora cordifolia* (Willd.) Miers ex Hook. f. & Thomson (Menispermaceae), a woody climber used in Bangladesh for the treatment of tuberculosis, cough and fever (Jahan et al. 2010). This phytoconstituent not only inhibits the growth of Gram-positive cocci *Streptococcus agalactiae* (Streptococcaceae) (Peng et al. 2015), but enhances the sensitivity of *Staphylococcus* strains towards antibiotics (Wojtyczka et al. 2014). In addition, medicinal plants produce inhibitors of bacterial resistance (Stermitz et al. 2000). Essential oil of coriander increases the sensitivity of *Acinetobacter baumannii* to tetracycline (Duarte et al. 2012). During the last few decades, scientists from all over the world are paying much more attention to the studies of an emerging branch of science, ethnobiology, especially to tribal medicine or ethnomedicine. Since 1980s, Bangladesh with 5500 plant species and more than 100 tribal communities belonging to over a dozen linguistic groups residing in various parts of the country with diversified plant species, varied culture, and a rich traditional knowledge system, possess an ethnobotanical emporia. Due to living close to nature, the tribal communities are custodians of an unique traditional knowledge system about ambient flora, fauna, and a rich heritage of phytomedicine or ethnomedicine. Since most of these ethnic communities do not have their written scripts and language, the information about prescriptions, pharmacology, attitude towards diseases, diagnosis, etc., of the age-old tribal medicines is lying unclaimed. The people relating to advanced societies are not aware of this rich knowledge system. A country like Bangladesh has many tropical rainforest plants rich with medicinal values (Rahmatullah et al. 2010). The Khyang tribe lives in a remote area and no reports exist on their medicinal plant use. In this context, we examined the antibacterial properties of medicinal plants used by Khyang tribe in Bangladesh by broth microdilution, time-killing and synergistic interaction assay. The aims of our study were: (i) to examine antibacterial properties of 18 medicinal plants of Bangladesh towards a panel of human pathogenic bacteria, (ii) to examine the antibacterial property of at least one major phytoconstituent from the most active plant, (iii) to determine the mode of action (i.e., bacteriostatic or bactericidal) of this phytoconstituent and (iv) and to determine the effect of the phytoconstituent on the sensitivity of MRSA to vancomycin and cefotaxime. The ultimate goal of our study is to contribute to the development of safe, effective and inexpensive plant-based materials to improve the current treatment strategies for bacterial infections.

## Materials and methods

### Medicinal plants collection

**Figure 1.**
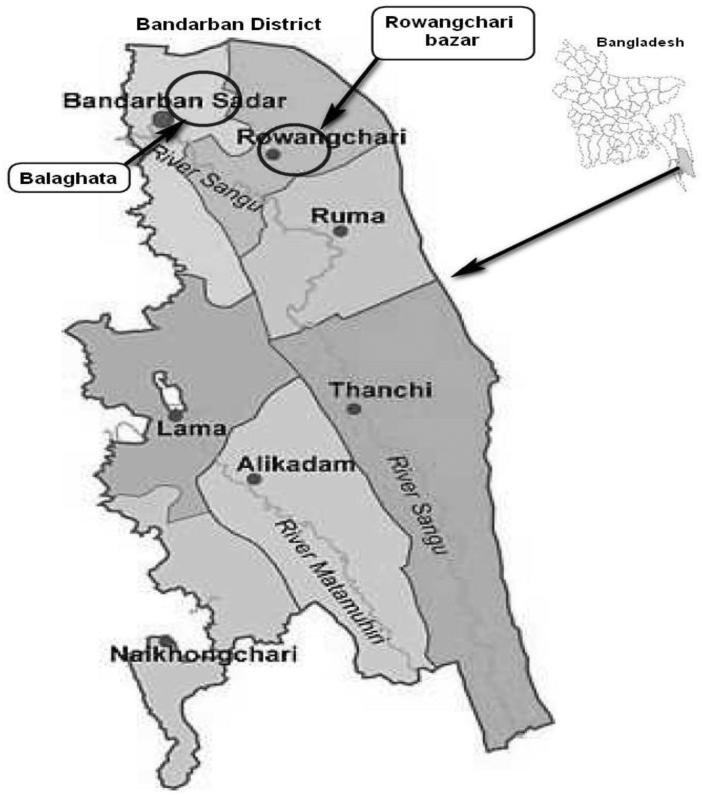
Map of Bandarban district and survey areas (circled).

A two-month survey for evaluation and documentation of the use of medicinal plants used for the day-to-day treatment of common diseases by traditional healers in Khyang tribe Bangladesh was performed from 5–30 April 2015 according to ethnopharmacological criteria (Cotton 1996). The survey was done on the Khyang tribe residing in villages adjoining Rowangchari bazar and Balaghata village in Bandarban district, Chittagong Hill Tracts, Bangladesh (Figure 1). Information gathered allowed the collection of 18 medicinal plants (Table 1), which were identified by Professor M. Atique Rahman, University of Chittagong. Voucher herbarium specimens with vernacular names, collecting localities, and dates of collections were deposited at the Medicinal Plants Collection Wing, Department of Pharmacy, University of Development Alternative, Dhaka, Bangladesh. After screening of superfluous matter, the collected leaves, bark, roots, rhizomes, seeds or fruits were separated and air-dried at room temperature for 2 weeks. The dried materials were then finely pulverized by grinding using aluminium collection blender (Philips, Shanghai, China) and the powders obtained were weighted with top loading balance (Sartorius AG, Göttingen, Germany).

**Table 1.** Traditional therapeutic properties of 18 medicinal plants from Bangladesh.

Family, genus species and authority	Voucher no.	Date of collection	Locality	Common name	Part used	Traditional therapeutic use
Apiaceae:						
*Coriandrum sativum* L.	030	14 April 2015	Balaghata	Dhone	Seeds	Gastrointestinal disorders
Brassicaceae:						
*Brassica alba* (L.) Rabenh.	022	13 April 2015	Balaghata	Haludsarisha	Seeds	Viral infections
*Lepidium sativum* L.	011	20 April 2015	Rowanchari bazar	Halimdana	Seeds	Viral infections
Combretaceae:						
*Terminalia bellirica* (Gaern.) Roxb.	006	19 April 2015	Rowanchari bazar	Bohera	Fruits	Fever, cough, dysentery, diarrhoea
Illiciaceae:						
*Illicum verum* Hook.f.	025	18 April 2015	Rowanchari bazar	ChakroPhool	Fruits	Flatulence
Lamiaceae:						
*Hyptis suaveolens* (L.) Poit.	001	25 April 2015	Rowanchari bazar	Tukma	Seeds	Gonorrhoea, fever, headache,
*Mentha arvensis* L.	014	26 April 2015	Rowanchari bazar	Pudinapata	Leaves	Diarrhoea, thrush
*Ocimum basilicum* L.	020	27 April 2015	Rowanchari bazar	Tulshibij	Leaves	Cold, coughs, viral or bacterial infection
*Vetiveria zizanioides* (L.) Nash	002	09 April 2015	Balaghata	Khoskhos	Roots	Bacterial infection, fever
Lauraceae:						
*Cinnamomum cassia* (L.) J. Presl.	035	18 April 2015	Rowanchari bazar	Toz	Bark	Nausea, vomiting and flatulence
Myristicaceae:						
*Myristica fragrans* Houtt.	063	19 April 2015	Rowanchari bazar	Joyfol biz	Fruits	Fever
Pedaliaceae:						
*Sesamum indicum* L.	021	26 April 2015	Rowanchari bazar	Sadatil	Seeds	Watery discharge from pregnant women
Piperaceae:						
*Piper nigrum* L.	057	20 April 2015	Rowanchari bazar	Sadagulmorich	Fruits	Fever, coughs, diarrhoea and diabetes
Ranunculaceae:						
*Nigella sativa* L.	005	8 April 2015	Balaghata	Kalojira	Seeds	Pain during menstruation, diabetes
Zingiberaceae:						
*Curcuma caesia* Roxb.	042	14 April 2015	Balaghata	Kala haila	Rhizomes	Tonsillitis
*Curcuma longa* L.	046	12 April 2015	Balaghata	Kalohalud	Rhizomes	Skin infection
*Curcuma pseudomontana* J. Graham	051	13 April 2015	Balaghata	Pahari Halud	Rhizomes	Cold
*Curcuma aeruginosa* Roxb.	052	13 April 2015	Balaghata	Kathaliholud	Rhizomes	Diarrhoea

### Medicinal plants extraction

The plant powders (20–60 g) were mixed at room temperature sequentially with organic solvents of increasing polarity starting with hexane (Friendemann Schmidt, Parkwood, Australia), ethyl acetate (Friendemann Schmidt, Parkwood, Australia) and 95% (v/v) ethanol (AR grade, John Kollin Corporation, Midlothian, UK) for differential extraction of non-polar, mid-polar and polar extracts, respectively (Harborne 1998). Each extraction was performed in triplicate by maceration of plant powder-to-solvent ratio of 1:5 (w/v) for three days at room temperature. The respective liquid extracts were subsequently filtered through qualitative filter papers No. 1 (Whatman International Ltd., Maidstone, UK) using aspirator pump (EW-35031-00, 18 L/min, 9.5 L Bath, 115 VAC), and the filtrates were concentrated to dryness under reduced pressure at 40 °C using rotary evaporator (Buchi Labortechnik AG, Flawil, Switzerland). The dry extracts obtained were weighed with an analytical balance (Sartorius AG, Göttingen, Germany) and stored in tightly closed glass scintillation vials (Kimble, Rockwood, TN) at −20 °C until further use. For stock solutions, each crude extract was dissolved in 100% dimethyl sulphoxide (DMSO) (R&M Chemicals, Chelmsford, UK) to a concentration of 100 µg/µL. A yield for each extract was calculated.

### Tested bacterial strains

Stock cultures of bacteria used for this study were kindly provided by the Department of Medical Microbiology, Faculty of Medicine, University of Malaya. The following human pathogenic bacteria were used as tested organisms: Gram positive organisms MRSA (University of Malaya Hospital clinical isolate), *Enterococcus faecalis* (ATCC 29212) and Gram negative organisms *Escherichia coli* (ATCC 25922), *Pseudomonas aeruginosa* (ATCC 15442), *Klebsiella pneumoniae* (University of Malaya Hospital clinical isolate) and *Acinetobacter baumannii* (University of Malaya Hospital clinical isolate).

### Phytoconstituents and control antibiotics

Cinnamaldehyde, eugenol, gallic acid, rifampicin, vancomycin and cefotaxime were purchased from Sigma-Aldrich (St. Louis, MO, >98% purity).

### Broth microdilution assay

Determination of minimum inhibitory concentration (MIC) was performed according to the Clinical and Laboratory Standards Institute guidelines (CLSI 2012). Briefly, bacterial strains were grown for 18–24 h at 37 °C. Direct suspension of the colonies were made in cationically adjusted Müeller-Hinton broth (CAMHB) and adjusted to OD_625_ 0.08–0.1 which corresponds to 1–2 × 10^8^ CFU/mL followed by serial 10-fold dilutions to give 1 × 10^6^ CFU/mL. Bacterial suspension (50 µL) was added to 96-well round bottom microtiter plates containing an equal volume of extracts or phytoconstituents at different concentrations and the 96-well plates were incubated for 24 h at 37 °C. The MIC is defined as the lowest concentration of material tested that completely inhibits the growth of bacteria. Minimum bactericidal concentration (MBC) was determined by sub-culturing the test dilutions on to a sterile agar plate and incubated further for 18–24 h. The highest dilution that yielded 0% bacterial growth on agar plates was taken as MBC. Both MIC and MBC values were calculated as the mean of triplicate experiments. Vancomycin and rifampicin were used as positive control antibiotics.

### Time-killing assay

Time-killing assay was conducted according to Giacometti et al. (1999). Bacteria (1 × 10^6^ CFU/mL) were incubated with cinnamaldehyde, eugenol, vancomycin or cefotaxime at 1 × MIC in Müeller-Hinton broth (MHB) at 37 °C. Bacterial suspensions (10 μL) were removed at various time intervals (1, 2, 3, 4 and 5 h), serially diluted in PBS, and plated onto Müeller-Hinton agar with 20–24 h at 37 °C to obtain viable colonies. Bacteria count Log_10_ values were calculated as the mean of triplicate experiments.

### Synergistic interaction assay

The ability of the hexane extract of bark of *Cinnamomum cassia* (L.) J. Presl. (Lauraceae), cinnamaldehyde and eugenol to increase the sensitivity of MRSA towards cefotaxime or vancomycin was measured by fractional inhibitory concentration (FIC) indices (FICIs) (Giacometti et al. 1999). Vancomycin was selected because the resistance of Gram-positive bacteria to this glycopeptide is a source of concern for clinicians (Courvalin 2006). FICI is the sum of the FIC of compound and FIC of antibiotic calculated according to the following formula (Berenbaum 1978):

$$\text{FIC }(\text{compound})\;=\;\frac{\text{MIC }(\text{compound in the presence of antibiotic})}{\text{MIC }(\text{compound alone})}$$

$$\text{FIC }\left(\text{antibiotic}\right)=\frac{\text{MIC }(\text{antibiotic in the presence of compound})}{\text{MIC }(\text{antibiotic alone})}$$

FICI = FIC (compound) + FIC (antibiotic)

The FICI results are interpreted as such: ≤0.5 synergistic, 0.5–1 additive, 1–4 indifferent; ≥4 antagonistic (Schelz et al. 2006).

## Results and discussion

### Medicinal plants collection

Survey for evaluation and documentation of the use of medicinal plants used in day-to-day practice by Khyang tribe residing in villages adjoining Rowangchari bazar and Balaghata village in Bandarban district, Chittagong Hill Tracts, Bangladesh (Figure 1) conducted from 5–30 April 2015 afforded the collection of 18 plants from 11 different families (Table 1). Twelve medicinal plants out of 18 were used to treat infections and all these belong to families known to accumulate essential oils except *Terminalia bellirica* (Gaern.) Roxb. (Combretaceae) (Takhtajan 2009). The ability of plants to synthesize and accumulate essential oils is not omnipresent in plants but scattered throughout the plant kingdom in certain families (Baser and Buchbauer 2015). Kar and Jain (1971) suggested that most of the anti-infectious traditional properties of aromatic plants enlisted in indigenous system of medicine are due to their essential oil contents. Essential oils are antibacterial (Deans and Ritchie 1987).

### Percentage yields

The yields of extracts were calculated using the following formula:

$$\text{\% yield}=\frac{\text{Mass of dried extract}}{\text{Mass of dried plant part}}\times \text{1}00$$

Dried plant parts were successively extracted with hexane, ethyl acetate and ethanol to obtain lipophilic (non-polar), amphiphilic (mid-polar) and hydrophilic (polar) extracts, respectively (Harborne 1998). The average yield values ranged from 2.3 to 10.8% indicating good extraction process (Parthasarathy et al. 2008) (Table 2). Calculated averages yields for hexane, ethyl acetate and ethanol extracts were 10.8, 3.6 and 2.3%, respectively. Hexane extracts had the highest average extraction yields confirming the predominance of lipophilic natural products in the plant parts extracted (Harborne 1998).

**Table 2.** Percentage yields (w/w).

		Percentage yield (%)		
Family, genus species	Part extracted	Hexane	Ethyl acetate	Ethanol
Apiaceae:				
*Coriandrum sativum*	Seeds	1.8	1.7	3.3
Brassicaceae:				
*Brassica alba*	Seeds	29.6	6.7	2.0
*Lepidium sativum*	Seeds	16.4	2.9	3.6
Combretaceae:				
*Terminalia bellirica*	Fruits	0.2	0.4	0.1
Illiciaceae:				
*Illicum verum*	Fruits	15.0	5.0	2.0
Lamiaceae:				
*Hyptis suaveolens*	Seeds	10.3	2.3	0.9
*Mentha arvensis*	Leaves	1.3	1.9	2.9
*Ocimum basilicum*	Leaves	9.8	1.7	0.5
*Vetiveria zizanioides*	Roots	0.6	1.7	0.1
Lauraceae:				
*Cinnamomum cassia*	Bark	0.9	1.8	10.8
Myristicaceae:				
*Myristica fragrans*	Fruits	33.9	10.9	3.3
Pedaliaceae:				
*Sesamum indicum*	Seeds	34.9	9.4	0.8
Piperaceae:				
*Piper nigrum*	Fruits	2.4	2.6	2.1
Ranunculaceae:				
*Nigella sativa*	Seeds	29.4	5.5	4.3
Zingiberaceae:				
*Curcuma caesia*	Rhizomes	2.6	2.3	1.2
*Curcuma longa*	Rhizomes	0.1	1.5	1.2
*Curcuma pseudomontana*	Rhizomes	0.7	2.6	2.4
*Curcuma aeruginosa*	Rhizomes	4.9	3.4	0.9
Average yield		10.8	3.6	2.3

### Broth microdilution assay

We sought to determine the MIC of 54 extracts from the 18 plants collected by broth microdilution method (Reller et al. 2009). Results of broth microdilution assay confirmed that Gram-positive bacteria were more susceptible than Gram-negative bacteria (Table 3). Rios and Recio (2005) suggested that crude extract with MIC superior to 1000 µg/mL is inactive and proposed interesting activity for MIC of 100 µg/mL and below. Fabry et al. (1998) defined active crude extracts as having MIC values below 8000 µg/mL. Kuete (2010) and Cos et al. (2006) use a stricter endpoint criteria, in which crude extracts with MIC values less than 100 µg/mL are active. Further, Kuete (2010) classifies as weakly active extracts with MIC above 625 µg/mL. Following Cos et al. (2006) and Kuete (2010), three plants had interesting activities with MIC below 100 µg/mL for at least one of the bacteria tested (Table 3). The lowest MIC towards MRSA was demonstrated by the hexane extract of *Mentha arvensis* L. (Lamiaceae) (24.3 µg/mL). According to Krishnan et al. (2010), antibacterial extracts or compounds are categorized into two classes: bacteriostatic (MBC/MIC ratio >4) and bactericidal (MBC/MIC ratio ≤4). Following this classification, hexane extract of *Mentha arvensis* with MBC/MIC ratio above 61.7 was bacteriostatic for MRSA; this extract was bacteriostatic for *E. coli* and bactericidal for *A. baumannii*. A body of experimental evidence demonstrates that it is not unusual for extracts to demonstrate equal MIC and MBC values. For instance, the ethanol extract of galls of *Quercus infectoria* Olivier (Fagaceae) inhibited the growth of MRSA with MIC and MBC values of 1600 µg/mL (Wan et al. 2014). Ethyl acetate extract of *Mentha piperita* L. (Lamiaceae) inhibited the growth of *E. faecalis* with MIC and MBC values of 2.5 mg/mL (Shalayel et al. 2017). The ethanol extract of *Terminalia bellirica* was strongly bactericidal for *A. baumannii* with MIC and MBC of 11.7 µg/mL. Hexane extract of bark of *Cinnamomum cassia* had the broadest spectrum of activity with notably a bactericidal effect for *A. baumannii* with MIC of 11.7 µg/mL.

**Table 3.** Minimum inhibitory concentrations (MIC) by broth microdilution assay.

			Mean MIC (µg/mL)^a^					
Family, genus species	Part extracted	Extract^b^	MRSA^c^	E.f^c^	E.c^c^	P.a^c^	K.p^c^	A.b^c^
Apiaceae:								
*Coriandrum sativum* seeds		E	375	375	187.5	187.5	187.5	375
Brassicaceae:								
*Brassica alba*	Seeds	H	375	187.5	750	>1500	375	375
		EA	1500	750	375	>1500	375	375
Combretaceae:								
*Terminalia bellirica*	Fruits	EA	187.5	>1500	187.5	>1500	187.5	375
		E	93.7/187.5^d^	375	23.4/750^d^	187.5	93.7/750^d^	11.7/11.7^d^
Illiciaceae:								
*Illicum verum*	Fruits	H	375	750	750	>1500	187.5	375
		EA	>1500	1500	375	>1500	1500	187.5
		E	187.5	187.5	275	>1500	187.5	375
Lamiaceae:								
*Hyptis suaveolens*	Seeds	H	375	187.5	187.5	375	375	375
*Mentha arvensis*	Leaves	H	24.3/>1500^d^	187.5	11.7/375^d^	375	187.5	93.7/187.5^d^
		EA	375	187.5	187.5	>1500	187.5	187.5
		E	275	187.5	375	>1500	750	375
*Ocimum basilicum*	Leaves	H	187.5	750	375	>1500	375	1500
*Vetiveria zizanioides*	Roots	H	375	187.5	187.5	>1500	>1500	750
Lauraceae:								
*Cinnamomum cassia*	Bark	H	46.8/187.5^d^	46.8/375^d^	46.8/93.8^d^	93.8/93.8^d^	46.8/375^d^	11.7/46.8^d^
		EA	375	187.5	750	1500	750	1500
		E	187.5	375	187.5	>1500	187.5	187.5
Myristicaceae:								
*Myristica fragrans*	Fruits	H	187.5	750	375	>1500	375	187.5
		EA	750	187.5	750	>1500	1500	187.5
		E	375	750	750	>1500	1500	750
Pedaliaceae:								
*Sesamum indicum*	Seeds	H	375	375	375	>1500	375	375
Piperaceae:								
*Piper nigrum*	Fruits	H	375	187.5	375	>1500	1500	1500
		EA	375	187.5	1500	>1500	1500	750
Zingiberaceae:								
*Curcuma longa*	Rhizomes	H	750	>1500	375	>1500	375	375
Positive control antibiotics:								
Vancomycin		1500/1500		11.7/11.7	n.t.	n.t.	n.t.	n.t.
Rifampicin		n.t.		n.t.	11.7/11.7	11.7/11.7	11.7/11.7	1500/1500

a Values are given as mean of triplicate. n.t.: not tested.

b H: hexane; EA: ethyl acetate; E: ethanol. Extracts with MIC > 625 µg/mL for all six bacteria tested are not included in the table (Kuete 2010).

c MRSA: methicillin-resistant *Staphylococcus aureus*; E.f: *Enterococcus faecalis*; E.c: *Escherichia coli*; P.a: *Pseudomonas aeruginosa*; K.p: *Klebsiella pneumoniae*; A.b: *Acinetobacter baumannii*.

d Minimum bactericidal concentrations (MBC) were determined for extracts with MIC < 100 µg/mL. Values are given as mean of triplicate.

Cinnamaldehyde is the major constituent of essential oil of *Cinnamomum cassia* bark (Tisserand and Young 2013), which also contains some eugenol (about 10%) (Lockwood 1979). Eugenol is also a component of *Mentha arvensis* (Vivek et al. 2009). Gallic acid is a major constituent of *Terminalia bellirica* (Latha and Daisy 2011). The antibacterial potency of these phytoconstituents was quantitatively examined by broth dilution method (Table 4). Rios and Recio (2005) suggested that MIC superior to 100 µg/mL for phytoconstituent was to be avoided because it is mildly active and proposed interesting activity with MIC of 10 µg/mL and below. According to Kuete (2010), the antibacterial activity of pure compounds is classified into three categories: MIC < 10 µg/mL: high; MIC between 10 and 100 µg/mL: medium and low for MIC above 100 µg/mL. Following both these classifications, eugenol with an MIC of 11.7 µg/mL and MIC/MBC ratios of 1.0 was strongly bactericidal against *E. faecalis*, *E. coli* and *K. pneumoniae*. Cinnamaldehyde was strongly bactericidal for *E. faecalis.* Ooi et al. (2006) tested the essential oil of *Cinnamomum cassia* bark and its major constituent cinnamaldehyde against a panel of bacteria and recorded activity against *S. aureus* and *P. aeruginosa* with MIC ranging from 75 to 600 µg/mL. Cinnamaldehyde, eugenol and gallic acid were moderately bactericidal for MRSA. Plant phenols, including eugenol are known for their membrane-disturbing activities (Sikkema et al. 1995). This mechanism of activity could at least account for the antibacterial properties of gallic acid (Smith et al. 2005; Borges et al. 2013). The different spectrum of activity between cinnamaldehyde and eugenol could at least be explained by the fact that polar antibacterial agents can pass the outer membrane through porin channels, whereas the outer membrane serves as a penetration barrier towards macromolecules (like vancomycin) and to non-polar compounds, and it is for this reason that Gram-negative bacteria are relatively resistant to non-polar molecules (Nikaido and Vaara 1985).

**Table 4.** Minimum inhibitory concentrations (MIC) and minimum bactericidal concentrations (MBC) of three phytoconstituents by broth microdilution assay.

		Mean MIC/MBC (µg/mL)^a^					
Phytoconstituent	Plant of origin	MRSA^b^	E.f^b^	E.c^b^	P.a^b^	K.p^b^	A.b^b^
Cinnamaldehyde	*Cinnamomum cassia*	750/1125	11.7/11.7	23.4/23.4	1500/1500	750/1125	1500/1500
Eugenol	*Cinnamomum cassia*	750/1125	11.7/11.7	11.7/11.7	23.4/23.4	11.7/11.7	1500/1500
	*Mentha arvensis*						
Gallic acid	*Terminalia bellirica*	750/1500	1500/1500	750/750	750/750	750/750	1500/1500
Positive control antibiotics:							
Vancomycin		1500/1500	11.7/11.7	n.t.	n.t.	n.t.	n.t.
Rifampicin		n.t.	n.t.	11.7/11.7	11.7/11.7	11.7/11.7	1500/1500

a Values are given as mean of triplicate. n.t.: not tested.

b MRSA: methicillin-resistant *Staphylococcus aureus*; E.f: *Enterococcus faecalis*; E.c: *Escherichia coli*; P.a: *Pseudomonas aeruginosa*; K.p: *Klebsiella pneumoniae*; A.b: *Acinetobacter baumannii*.

### Time-killing assay

Cinnamaldehyde and eugenol were tested against MRSA for time-killing assay as this bacterium represents the greatest current medical need (Ling et al. 2015). The result of time-killing assay is presented in Table 5. A perusal of this table shows that cinnamaldehyde (4 × MIC) and eugenol at both 2 and 4 × MIC evoked at 1 h a fall of Log_10_ (CFU/mL) bacteria count from 6 to 0. This effect was permanent confirming bactericidal activity. Gill and Holley (2006) made the demonstration that cinnamaldehyde at high concentration was bactericidal on *E. coli* via inhibition of membrane-bound ATPase activity. This small molecular weight molecule being lipophilic may penetrate and destabilize the cytoplasmic membrane of MRSA leading to nutrients and energy depletion (Sikkema et al. 1995). In previous study, cinnamaldehyde was inhibitory for the growth of the enteric bacteria but exhibited neither outer membrane-disintegrating activity nor depletion of intracellular ATP (Helander et al. 1998). In addition, aldehyde group conjugated to a carbon to carbon double bond is a highly electronegative arrangement, which may explain the observed activity (Moleyar and Narasimham 1986). Such electronegative compounds may interfere in biological processes involving electron transfer and reaction with vital nitrogen components, e.g., proteins and nucleic acids, and therefore inhibit the growth of the microorganisms. Cinnamaldehyde may also bind to amino acids in enzymes via its carbonyl group (Wendakoon and Sakaguchi 1993).

**Table 5.** Time-killing assay of eugenol and cinnamaldehyde against methicillin-resistant *Staphylococcus aureus*.

	Bacteria count Log10 (CFU/mL)^a^					
Time (h)	0	1	2	3	4	5
Eugenol (2 × MIC)	6	0	0	0	0	0
Eugenol (4 × MIC)	6	0	0	0	0	0
Cinnamaldehyde (2 × MIC)	6	4.6	4.5	4.0	0	0
Cinnamaldehyde (4 × MIC)	6	0	0	0	0	0
Cefotaxime (1 × MIC)	6	4.7	4.1	4.0	4.0	4.1
Vancomycin (1 × MIC)	6	4.4	3.4	3.6	3.1	3.1

a Values are given as mean of triplicate.

### Synergistic interaction assay

Cinnamaldehyde has been reported to be synergistic with ampicillin, penicillin, tetracycline or novobiocin against *E. coli* (Palaniappan and Holley 2010). Eugenol is a constituent of *Cinnamomum cassia* bark. In this context, we sought to determine the synergy effects of the hexane extract of *Cinnamomum cassia* bark, cinnamaldehyde and eugenol with vancomycin and cefotaxime towards MRSA (Table 6). Both extract and cinnamaldehyde did not increase the sensitivity of MRSA to cefotaxime. However, we observed that cinnamaldehyde has a high synergistic effect with vancomycin with an FICI of 0.3. We do not know by which mechanism cinnamaldehyde increases the sensitivity of MRSA to vancomycin. Eugenol with FICI above 4 was antagonistic for both antibiotics. Bacteria resist vancomycin by mutating a gene coding for terminal d-Ala-d-Ala in the peptidoglycan wall resulting in Ala-d-Lac resulting in 1000 decreased affinity of vancomycin (Walsh 2000). Wright (2000) proposed to develop agents ‘resisting’ resistance as a strategy to fight superbugs and cinnamaldehyde showing no unreasonable adverse effects to humans (Cocchiara et al. 2005) is an exciting candidate. Only with exact knowledge of the mechanisms underlying the synergy effect observed, it will be possible to develop a new generation of safe and standardized with high efficacy (Wagner and Ulrich-Merzenich 2009).

**Table 6.** Fractional inhibitory concentration index (FICI) of different combination of hexane extract of *Cinnamomum cassia*, cinnamaldehyde or eugenol, and antibiotics against methicillin-resistant *Staphylococcus aureus*.

	FICI^a^	Effect^b^
Extract^c^		
Cefotaxime	>4.2	Antagonistic
Vancomycin	>4.2	Antagonistic
Cinnamaldehyde		
Cefotaxime	2.2	Indifferent
Vancomycin	0.3	Synergistic
Eugenol		
Cefotaxime	>4.2	Antagonistic
Vancomycin	>4.2	Antagonistic

a Values are given as mean of triplicate.

b FICI: ≤0.5 synergistic, 0.5–1 additive, 1–4 indifferent; ≥4 antagonistic (Schelz et al. 2006).

c Hexane extract of bark of *Cinnamomum cassia*.

## Conclusions

Over the past few decades, there has been a dramatic decrease in the number of new antibiotic approved by the FDA. MRSA is a cause for concern due to the small number of antibiotics effective against this organism and resistance associated with their uses. The development of resistant-modifying agents could be a supplemental strategy to overcome resistance. The current result shows that *Cinnamomum cassia* has a broad-spectrum antibacterial activity. From this plant, cinnamaldehyde is a resistant-modifying agent that decreases the resistance of MRSA to vancomycin. Our study provides evidence that the medicinal plants in Bangladesh have high potential for the development of plant-based material to improve the current treatment strategies for bacterial infections.

## References

[CIT0001] Alanis AJ. 2005 Resistance to antibiotics: are we in the post-antibiotic era? Arch Med Res. 36:697–705. 16216651 10.1016/j.arcmed.2005.06.009

[CIT0002] Baser KHC, Buchbauer G. 2015 Handbook of essential oils: science, technology, and applications. Boca Raton (FL): CRC Press.

[CIT0003] Berenbaum MC. 1978 A method for testing for synergy with any number of agents. J Infect Dis. 137:122–130. 627734 10.1093/infdis/137.2.122

[CIT0004] Borges A, Ferreira C, Saavedra MJ, Simoes M. 2013 Antibacterial activity and mode of action of ferulic and gallic acids against pathogenic bacteria. Microb Drug Resist. 19:256–265. 23480526 10.1089/mdr.2012.0244

[CIT0005] Bratu S, Landman D, Haag R, Recco R, Eramo A, Alam M, Quale J. 2005 Rapid spread of carbapenem-resistant *Klebsiella pneumoniae* in New York City: a new threat to our antibiotic armamentarium. Arch Intern Med. 165:1430–1435. 15983294 10.1001/archinte.165.12.1430

[CIT0006] Charles PG, Grayson ML. 2004 The dearth of new antibiotic development: why we should be worried and what we can do about it. Med J Aus. 181:549–553. 10.5694/j.1326-5377.2004.tb06444.x 15540967

[CIT0007] Clinical and Laboratory Standards Institute 2012 Methods for dilution antimicrobial susceptibility tests for bacteria that grow aerobically; approved standard. 9th ed CLSI document M07-A9. Wayne (PA): Clinical and Laboratory Standards Institute.

[CIT0008] Cocchiara J, Letizia CS, Lalko J, Lapczynski A, Api AM. 2005 Fragrance material review on cinnamaldehyde. Food Chem Toxicol. 43:867–923. 15811572 10.1016/j.fct.2004.09.014

[CIT0009] Cos P, Vlietinck AJ, Vanden Berghe D, Maes L. 2006 Anti-infective potential of natural products: how to develop a stronger in vitro ‘proof-of concept’. J Ethnopharmacol. 106:290–302. 16698208 10.1016/j.jep.2006.04.003

[CIT0010] Cotton CM. 1996 Ethnobotany: principles and applications. New York: John Wiley & Sons.

[CIT0011] Courvalin P. 2006 Vancomycin resistance in Gram-positive cocci. Clin Infect Dis. 42:S25–S34. 16323116 10.1086/491711

[CIT0012] Deans SG, Ritchie G. 1987 Antibacterial properties of plant essential oils. Int J Food Microbiol. 5:165–180.

[CIT0013] Duarte A, Ferreira S, Silva F, Domingues FC. 2012 Synergistic activity of coriander oil and conventional antibiotics against *Acinetobacter baumannii*. Phytomedicine. 19:236–238. 22240078 10.1016/j.phymed.2011.11.010

[CIT0014] Fabry W, Okemo PO, Ansorg R. 1998 Antibacterial activity of East African medicinal plants. J Ethnopharmacol. 60:79–84. 9533435 10.1016/s0378-8741(97)00128-1

[CIT0015] Fung-Tomc J, Huczko E, Pearce M, Kessler RE. 1988 Frequency of *in vitro* resistance of *Pseudomonas aeruginosa* to cefepime, ceftazidime, and cefotaxime. Antimicrob Agents Chemother. 32:1443–1445. 3143306 10.1128/aac.32.9.1443 PMC175888

[CIT0016] Giacometti A, Cirioni O, Barchiesi F, Fortuna M, Scalise G. 1999 *In vitro* anticryptosporidial activity of ranalexin alone and in combination with other peptides and with hydrophobic antibiotics. Eur J Clin Microbiol Infect Dis. 18:827–829. 10614961 10.1007/s100960050410

[CIT0017] Gill AO, Holley RA. 2006 Inhibition of membrane bound ATPases of *Escherichia coli* and *Listeria monocytogenes* by plant oil aromatics. Int J Food Microbiol. 111:170–174. 16828188 10.1016/j.ijfoodmicro.2006.04.046

[CIT0018] Goettsch W, Van Pelt W, Nagelkerke N, Hendrix MGR, Buiting AGM, Petit PL, Sabbe LJM, Van Griethuysen AJA, De Neeling AJ. 2000 Increasing resistance to fluoroquinolones in *Escherichia coli* from urinary tract infections in the Netherlands. J Antimicrob Chemother. 46:223–228. 10933644 10.1093/jac/46.2.223

[CIT0019] Harborne JB. 1998 Phytochemical methods: a guide to modern techniques of plant analysis. 3rd ed. London: Chapman and Hall.

[CIT0020] Helander IM, Alakomi HL, Latva-Kala K, Mattila-Sandholm T, Pol I, Smid EJ, Gorris LG, von Wright A. 1998 Characterization of the action of selected essential oil components on Gram-negative bacteria. J Agric Food Chem. 46:3590–3595.

[CIT0021] Jahan R, Khatun MA, Nahar N, Jahan FI, Chowdhury AR, Nahar A, Seraj S, Mahal MJ, Khatun Z, Rahmatullah M. 2010 Use of Menispermaceae family plants in folk medicine of Bangladesh. Adv Nat Appl Sci. 4:1–9.

[CIT0022] Jevons MP. 1961 “Celbenin”-resistant staphylococci. Br Med J. 1:124–125.

[CIT0023] Kar A, Jain SR. 1971 Antibacterial evaluation of some indigenous medicinal volatile oils. Qual Plant Mater Veg Food Nutr. 20:231–237.

[CIT0024] Krishnan N, Ramanathan S, Sasidharan S, Murugaiyah V, Mansor SM. 2010 Antimicrobial activity evaluation of *Cassia spectabilis* leaf extracts. Int J Pharmacol. 6:510–514.

[CIT0025] Kuete V. 2010 Potential of Cameroonian plants and derived products against microbial infections: a review. Planta Med. 76:1479–1491. 20533165 10.1055/s-0030-1250027

[CIT0026] Latha RCR, Daisy P. 2011 Insulin-secretagogue, antihyperlipidemic and other protective effects of gallic acid isolated from *Terminalia bellerica* Roxb. in streptozotocin-induced diabetic rats. Chem Biol Interact. 189:112–118. 21078310 10.1016/j.cbi.2010.11.005

[CIT0027] Ling LL, Schneider T, Peoples AJ, Spoering AL, Engels I, Conlon BP, Mueller A, Schäberle TF, Hughes DE, Epstein S, et al 2015 A new antibiotic kills pathogens without detectable resistance. Nature. 517:455–459. 25561178 10.1038/nature14098 PMC7414797

[CIT0028] Lockwood GB. 1979 The major constituents of the essential oils of *Cinnamomum cassia* Blume growing in Nigeria. Planta Med. 36:380–381.

[CIT0029] MacKenzie FM, Forbes KJ, Dorai-John T, Amyes SGB, Gould IM. 1997 Emergence of a carbapenem-resistant *Klebsiella pneumoniae*. Lancet. 350:783–783. 9298003 10.1016/s0140-6736(05)62567-6

[CIT0030] Moleyar V, Narasimham P. 1986 Antifungal activity of some essential oil components. Food Microbiol. 3:331–336. 10.1016/0168-1605(92)90035-2 1457292

[CIT0031] Neu HC. 1992 The crisis in antibiotic resistance. Science (New York, N.Y.). 257:1064–1074. 10.1126/science.257.5073.1064 1509257

[CIT0032] Nikaido H, Vaara M. 1985 Molecular basis of bacterial outer membrane permeability. Microbiol Rev. 49:1–32. 2580220 10.1128/mr.49.1.1-32.1985 PMC373015

[CIT0033] O’Neill J. 2014. Tackling drug-resistant infections globally: final report and recommendations. UK: HM Government and Welcome Trust.

[CIT0034] Ooi LS, Li Y, Kam SL, Wang H, Wong EY, Ooi VE. 2006 Antimicrobial activities of cinnamon oil and cinnamaldehyde from the Chinese medicinal herb *Cinnamomum cassia* Blume. Am J Chin Med. 34:511–522. 16710900 10.1142/S0192415X06004041

[CIT0035] Palaniappan K, Holley RA. 2010 Use of natural antimicrobials to increase antibiotic susceptibility of drug resistant bacteria. Int J Food Microbiol. 140:164–168. 20457472 10.1016/j.ijfoodmicro.2010.04.001

[CIT0036] Parthasarathy VA, Chempakam B, Zachariah TJ. 2008 Chemistry of spices. Wallingford (UK): CAB International.

[CIT0037] Peleg AY, Seifert H, Paterson DL. 2008 *Acinetobacter baumannii*: emergence of a successful pathogen. Clin Microbiol Rev. 21:538–582. 18625687 10.1128/CMR.00058-07 PMC2493088

[CIT0038] Peng L, Kang S, Yin Z, Jia R, Song X, Li L, Li Z, Zou Y, Liang X, Li L, et al 2015 Antibacterial activity and mechanism of berberine against *Streptococcus agalactiae*. Int J Clin Exp Pathol. 8:5217–5223. 26191220 PMC4503092

[CIT0039] Rahmatullah M, Hasan ME, Islam MA, Islam MT, Jahan FI, Chowdhury AR, Jamal F, Islam MS, Miajee Z, Jahan R, et al 2010 A survey on medicinal plants used by the folk medicinal practitioners in three villages of Panchagarh and Thakurgaon district, Bangladesh. Am-Eur J Sustain Agric. 4:291–301.

[CIT0040] Reller LB, Weinstein M, Jorgensen JH, Ferraro MJ. 2009 Antimicrobial susceptibility testing: a review of general principles and contemporary practices. Clin Infect Dis. 49:1749–1755. 19857164 10.1086/647952

[CIT0041] Rios JL, Recio MC. 2005 Medicinal plants and antimicrobial activity. J Ethnopharmacol. 100:80–84. 15964727 10.1016/j.jep.2005.04.025

[CIT0042] Schelz Z, Molnar J, Hohmann J. 2006 Antimicrobial and antiplasmid activities of essential oils. Fitoterapia. 77:279–285. 16690225 10.1016/j.fitote.2006.03.013

[CIT0043] Shalayel MHF, Asaad AM, Qureshi MA, Elhussein AB. 2017 Anti-bacterial activity of peppermint (*Mentha piperita*) extracts against some emerging multi-drug resistant human bacterial pathogens. J Herbal Med. 7:27–30.

[CIT0044] Sikkema J, De Bont JA, Poolman B. 1995 Mechanisms of membrane toxicity of hydrocarbons. Microbiol Rev. 59:201–222. 7603409 10.1128/mr.59.2.201-222.1995 PMC239360

[CIT0045] Smith AH, Zoetendal E, Mackie RI. 2005 Bacterial mechanisms to overcome inhibitory effects of dietary tannins. Microb Ecol. 50:197–205. 16222487 10.1007/s00248-004-0180-x

[CIT0046] Spellberg B, Powers JH, Brass EP, Miller LG, Edwards JE. 2004 Trends in antimicrobial drug development: implications for the future. Clin Infect Dis. 38:1279–1286. 15127341 10.1086/420937

[CIT0047] Stermitz FR, Lorenz P, Tawara JN, Zenewicz LA, Lewis K. 2000 Synergy in a medicinal plant: antimicrobial action of berberine potentiated by 5′-methoxyhydnocarpin, a multidrug pump inhibitor. Proc Natl Acad Sci USA. 97:1433–1437. 10677479 10.1073/pnas.030540597 PMC26451

[CIT0048] Stryjewski ME, Corey GR. 2014 Methicillin-resistant *Staphylococcus aureus*: an evolving pathogen. Clin Infect Dis. 58:S10–S19. 24343827 10.1093/cid/cit613

[CIT0049] Takhtajan A. 2009 Flowering plants. New York: Springer Science & Business Media.

[CIT0050] Tisserand R, Young R. 2013 Essential oil safety: a guide for health care professionals. Edinburgh (UK): Elsevier Health Sciences.

[CIT0051] Vivek S, Nisha S, Harbans S, Devendra SK, Vijaylata P, Bikram S, Raghbir GC. 2009 Comparative account on GC–MS analysis of *Mentha arvensis* L. (corn-mint) from three different locations of north India. Int J Drug Dev Res. 1:1–9.

[CIT0052] Wagner H, Ulrich-Merzenich G. 2009 Synergy research: approaching a new generation of phytopharmaceuticals. Phytomedicine. 16:97–110. 19211237 10.1016/j.phymed.2008.12.018

[CIT0053] Walsh C. 2000 Molecular mechanisms that confer antibacterial drug resistance. Nature. 406:775–781. 10963607 10.1038/35021219

[CIT0054] Wan NAW, Masrah M, Hasmah A, Noor IN. 2014 In vitro antibacterial activity of *Quercus infectoria* gall extracts against multidrug resistant bacteria. Trop Biomed. 31:680–688. 25776593

[CIT0055] Wendakoon CN, Sakaguchi M. 1993 Combined effect of sodium chloride and clove on growth and biogenic amine formation of *Enterobacter aerogenes* in mackerel muscle extract. J Food Prot. 56:410–413. 10.4315/0362-028X-56.5.410 31084146

[CIT0056] Wojtyczka RD, Dziedzic A, Kepa M, Kubina R, Kabala-Dzik A, Mularz T, Idzik D. 2014 Berberine enhances the antibacterial activity of selected antibiotics against coagulase-negative *Staphylococcus* strains *in vitro*. Molecules. 19:6583–6596. 24858093 10.3390/molecules19056583 PMC6272005

[CIT0057] World Health Organization (WHO) 2014 Antimicrobial resistance: 2014 global report on surveillance. Geneva: World Health Organization.

[CIT0058] Wright GD. 2000 Resisting resistance: new chemical strategies for battling superbugs. Chem Biol. 7:R127–R132. 10873842 10.1016/s1074-5521(00)00126-5

